# Is it truly impossible to strengthen the vastus medialis in isolation from the entire quadriceps muscle group?

**DOI:** 10.1016/j.heliyon.2024.e41012

**Published:** 2024-12-06

**Authors:** Seyyed Hossein Hosseini, Farzam Farahmand

**Affiliations:** a Department of Sports Sciences, University of Guilan, Rasht, Iran; b Department of Mechanical Engineering, Sharif University of Technology, Tehran, Iran; c Djavad Mowafaghian Research Center for Intelligent Neuro-Rehabilitation Technologies, Iran

**Keywords:** Vastus medialis, Quadriceps, Selective strengthening, Lateral patellar compression syndrome, Muscle activity, Cross-section

## Abstract

**Background:**

To date, no specific exercises have been designed to selectively strengthen the vastus medialis muscle. Therefore, the purpose of this research is to provide a clear answer to the question: Is it truly impossible to strengthen the vastus medialis independently of the entire quadriceps muscle group? Methods: Thirty-three females with lateral patellar compression syndrome were randomly divided into 2 groups: one focused on selective strengthening of the vastus medialis (n = 16) and the other on general strengthening of the quadriceps (n = 17). The intervention lasted 8 weeks. Muscles cross-sectional area and electrical activity were assessed using ultrasound and electromyography, respectively. Data analysis was done using independent and dependent t-tests.

**Results:**

In the selective strengthening group, significant increases were observed in the activity and cross-sectional area of the vastus medialis, as well as in the ratios of vastus medialis/vastus lateralis activity and cross-section from pre-to post-intervention (P < 0.01). In the general strengthening group, significant increases were noted in the cross-sectional area of the vastus lateralis and the activity of both muscles, with a greater increase in the vastus lateralis (P < 0.01).

**Conclusions:**

Contrary to previous evidence, our findings demonstrate that preferential activation and selective strengthening of the vastus medialis muscle, independent of the other quadriceps components, is achievable through the training protocol employed in this study.

## Highlights

• Traditional quadriceps exercises maintain or increase imbalance between vastus muscles.
• Our selective strengthening protocol eliminate imbalance between vastus muscles.
• This protocol allows selective strengthening of vastus medialis independent of quadriceps other components.

## 1 Introduction

Anterior knee pain encompasses various conditions, with patellofemoral pain being the most prevalent. This condition is often linked to changes in activity and underlying neuromuscular impairments [1]. It is a chronic issue frequently encountered by sports medicine clinicians, presenting challenges for both patients and practitioners due to its complex nature. Effective evaluation and management of factors such as altered biomechanics, muscular weakness, soft-tissue tightness, and neuromuscular activation patterns are crucial for appropriate treatment [2]. In many instances, compression in the lateral soft tissue of the knee and pressure on the lateral aspect of the patella lead to patellofemoral pain, a condition known as lateral patellar compression [3] or lateral patellar impingement [4] syndrome. Lateral patellar compression syndrome manifests as a series of clinical symptoms caused by abnormal increases in lateral patellofemoral joint pressure, often resulting from a long-standing lateral inclination of the patella without dislocation, adaptive shortening of the lateral retinaculum, and chronic stress imbalances between the internal and external articular surfaces [4,5]. Among the key factors contributing to dynamic imbalance are discrepancies in muscle activity [[6], [7], [8], [9]] and morphology [[10], [11], [12], [13]] between the medial and lateral parts of the quadriceps muscle.

The imbalance between the vastus medialis and vastus lateralis muscles can hinder the vastus medialis's ability to counteract the vastus lateralis, resulting in patellar maltracking and excessive lateral tracking of the patella, which induces pain. Researchers have identified both vastus medialis insufficiency [8,14] and vastus lateralis tightness [15] as significant contributors to patellofemoral joint disorders. Consequently, exercise therapy may include interventions to either strengthen the weakened vastus medialis or stretch the tight vastus lateralis muscle [15,16].

Based on this, researchers have attempted to design and suggest exercises that selectively strengthen the vastus medialis compared to the vastus lateralis, aiming to restore normal function in the patellofemoral joint [11,[17], [18], [19]]. However, they have not yet introduced an exercise that effectively engages and strengthens the vastus medialis more than the other components of the quadriceps muscle. Some researchers have argued against the possibility of selectively activating the vastus medialis over the vastus lateralis [20,21]. However, these studies primarily focus on pain and functional outcomes rather than on the strength and morphology of the vastus medialis and lateralis muscles, which are critically impacted in affected patients. Although both selective and general quadriceps strengthening exercises may lead to significant improvements in pain and function, it is unreasonable to assume they have equivalent effects on muscle activity and morphology.

A comprehensive muscular strengthening program must correct any strength imbalances between the medial and lateral quadriceps to restore the patellar biomechanical alignment [22]. If such an imbalance is not corrected, the effectiveness of therapeutic exercise programs is questionable, as the patella will remain misaligned and will be more prone to re-injury, even if the joint pain improves [7]. However, most traditional therapeutic approaches for improving patellofemoral pain focus on enhancing overall quadriceps strength without addressing the inherent imbalance between the vastus medialis and vastus lateralis muscles. This oversight may exacerbate lateral patellar displacement within the femoral groove, worsening this syndrome.

Also, the few previous studies aimed at selectively strengthening the vastus medialis have relied on indirect methods, such as electromyography, often yielding inconclusive results. For instance, Smith et al. (2009) noted in their systematic review that the vastus medialis cannot be preferentially activated simply by altering lower limb joint orientation or the addition of co-contraction [23]. The limitation of isolated electromyography is that it only assesses muscle activation, overlooking other critical factors affecting muscle force and mechanical impact on the patella, such as physiological cross-sectional area [24]. In this study, we aimed to measure essential parameters influencing the torque applied to the patella, including electromyography activity and cross-sectional area of the vastus medialis and lateralis muscles. Our primary objective is to answer whether it is genuinely impossible to strengthen the vastus medialis muscle selectively and independently from the quadriceps group. We hypothesize that, contrary to prior claims, the vastus medialis can be selectively strengthened through isokinetic knee extension exercises performed in the final 30 degrees of the range of motion, combined with maximum external rotation of the leg.

## 2 Materials and methods

### 2.1 Study design

This study was a randomized controlled trial conducted at Nourafshar Sports Medicine and Rehabilitation Hospital, between February 2021 and January 2022. Participants were randomly assigned in a 1:1 ratio to either the selective or general group using a computer-generated random allocation sequence with permuted blocks of varying sizes. Allocation was concealed using sequentially numbered, opaque, sealed envelopes prepared by personnel not involved in the trial. During the full trial period, outcome assessors were blinded to group allocation, and intervention providers were blinded to outcome assessments. To maintain blinding for the participants and conceal the study hypothesis, participants were not informed of the specific treatment methods in each group or the hypothesis being tested. The study adhered to the ethical standards of the World Medical Association Declaration of Helsinki, with ethical approval obtained from the Research and Technology Council of Nahavand University (Ethical Code IR. NAHGU.REC.1399.003).

### 2.2 Participants

The study included female patients with lateral patellar compression syndrome who met the inclusion criteria and agreed to participate. Written informed consent was obtained from all participants. Initially, 34 volunteer patients were selected using convenience sampling and randomly divided into two equal groups: The Selective and General groups. During the training programs, one patient from the Selective group withdrew, resulting in a final sample size of 33 participants—16 in the Selective group and 17 in the General group—for post-intervention measurements.

Because the etiology and mechanism of lateral patellar compression syndrome is complex and diverse, it is very difficult and controversial to accurately describe its clinical symptoms and establish definite diagnostic criteria. To date, no well-established reference for diagnosis exists [25]. Currently, its diagnosis mainly depends on the subjective judgment of clinicians and several examination results [4,[26], [27], [28]]; Therefore, a combination of criteria suggested by previous research and detailed examinations by an experienced physiotherapist were used to diagnose patients with this syndrome. Inclusion criteria were the persistent anterior knee pain aggravated by stress on the patellofemoral joint; the lateral margin or lateral retinaculum showing limited tenderness points on physical examination; positive patellar grinding and patella elution tests; lateral inclination of the patella, narrowed lateral patellofemoral joint space, contracture and tapering of the lateral retinaculum and degenerative changes in the patellofemoral articular cartilage, on the imaging examination [25]; positive the functional knee, patellar compression and patellar apprehension tests; age range from 18 to 30 years and the presence of patellofemoral pain symptoms in a period of more than 6 months [13]. Exclusion criteria were knee meniscus or ligamentous injuries; knee arthritis; history of patellar dislocation; history of physiotherapy or knee surgery; presence of neurological disorders such as defective atrial system; pregnancy of subjects; lack of normal BMI; and knee anterior bursitis [13]; malunion of patella fracture, free bodies in the joint, surgical contraindications, and bilateral knee involvement [25].

### 2.3 Instruments and measurements

This study was approved in February 2021 and concluded in 2022. Baseline measurements were taken on November 27, 2020. Participants then engaged in training programs from November 29, 2020 to January 21, 2021 for eight weeks. Post-intervention measurements were conducted on January 22, 2021.

#### 2.3.1 Muscle cross-sections

**Fig. 1:**
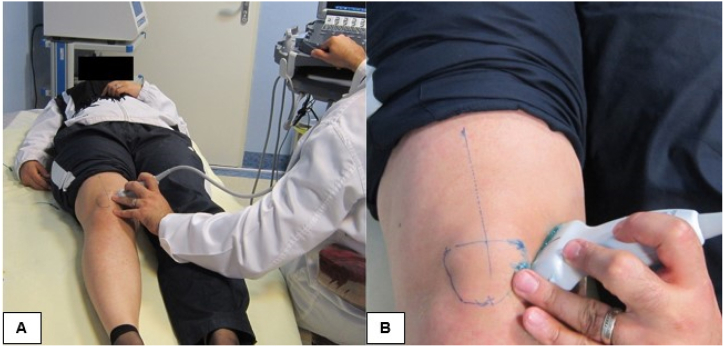
Muscle imaging technique with ultrasound. (a) The subjects were placed supinely on a bed with both lower limbs in neutral position and knees in full extension. (b) The medial (vastus medialis) and lateral (vastus lateralis) patellar aspects were scanned.

A two-dimensional digital ultrasound device (SonoSite M-Turbo, Inc., Bothell, WA, USA) equipped with a linear probe, was used to imaging the muscles. This device provides a high quality image with a very high resolution and contrast. The medial (vastus medialis) and lateral (vastus lateralis) aspects of the distal thigh, in the vicinity of the inner and outer patellar margins, respectively, were scanned under sterile conditions [29]. During the sonographic assessment, participants lay supine on a bed with their knees fully extended, and both lower limbs were positioned neutrally (Fig. 1a) with the help of a wall to prevent lower limb rotation [16]. When touching the ultrasound probe with the patients skin, minimal pressure was applied to ensure that the muscle did not lose its natural shape. Ultrasound's validity for assessing vastus muscle morphology is well-established [7]. Cross-sections were measured at the patella's base: for the vastus medialis, from the highest to the lowest attachment point on the medial patellar border; and for the vastus lateralis, from the highest to the lowest attachment point on the lateral border. Fig. 1b illustrates the scanning method for the vastus medialis. Muscle cross-sections were measured in square centimeters. The muscle cross-sections were selected by moving from the upper patellar border downward until they disappear [7].

#### 2.3.2 The muscles electromyography activities

To assess the electromyography activity of the vastus medialis and vastus lateralis muscles, an 8-channel surface electromyography device (P3X8, DataLog, Biometrics Ltd, Cwmfelinfach, Gwent, UK) with Ag-AgCl bipolar surface electrodes and a preamplifier (SX230, Biometrics Ltd, Cwmfelinfach, Gwent, UK) was used. After preparing the skin, the surface electrodes with a distance of 20 mm from each other's center were placed on the skin, approximately 4 cm higher and 3 cm inside than the superomedial corner of the patella at a 55-degree angle to the thigh's longitudinal axis (for the vastus medialis), and 10 cm above and 7 cm outside of the superolateral corner of the patella at a 15-degree angle to the thigh's longitudinal axis (for the vastus lateralis) [19]. The reference electrode was attached to the tibial tuberosity. Electrodes were secured with Velcro tape to prevent disconnection during movement. Signals were recorded at a 1000-Hz sampling rate, amplified with a bandwidth of 20–450 Hz (input impedance = 10^15^ Ω, common mode rejection ratio >110 dB, gain = 1000), and analyzed offline using a root mean square (RMS) filter (DataLog software; Biometrics Ltd).

EMG activity was recorded during concentric knee extension, starting from 45 degrees of knee flexion while seated, with the ankle in a neutral position. The extension was completed within 3 s, and the knee was held fully extended for 2 s at the end of this movement. This task was repeated three times with a 90-s interval, and the average RMS value of these three times was calculated. This value was normalized by dividing it by the average RMS from the three maximum voluntary isometric contractions (MVIC) of full knee extension and multiplying by 100. MVICs were performed before and after interventions, with subjects encouraged to contract the quadriceps maximally for 6 s, repeated three times with 90-s intervals.

### 2.4 Interventions

Participants in each group engaged in a 7-min warm-up before their exercises. Each group then performed their respective exercises: selective strengthening exercises for the vastus medialis (Selective group) and general quadriceps strengthening exercises (General group).

#### 2.4.1 Selective strengthening exercises of the vastus medialis

The training program for the Selective group focused on strengthening the vastus medialis. Exercises were performed using a Biodex isokinetic dynamometer (Biodex System 4 Pro multi-joint machine, USA). The protocol involved isokinetic knee extension exercises at speeds of 90, 120, and 150°/second, executed at a maximum external rotation of the lower leg from a reference position of 30 degrees of knee flexion. Each exercise consisted of a concentric-eccentric cycle. Participants were seated with a hip angle of 100° and a relatively upright trunk, while the dynamometer was aligned with the knee's rotation axis. This axis was defined at the end of the thigh, in line with the femoral condyles. Using specialized belts, the trunk and thigh were secured to the chair, while the lower leg was connected to the dynamometer arm slightly above the ankle. During knee extension, maximum torque was applied, and a strong band was used to maintain the maximum tolerable external rotation of the leg.

#### 2.4.2 General strengthening exercises of the quadriceps

The training program of the General group included overall quadriceps strengthening exercises, performed in a non-weight-bearing, supine position: The exercises consisted of:1) Maximal isometric contraction of the quadriceps with the knee fully extended; 2) Straight leg raises up to approximately 30 degrees of hip flexion with the knee straight; 3) Knee extensions through a small arc from 10 degrees of flexion to full extension. Each exercise was conducted in three sets of 10 repetitions, with a hold of 6 s per repetition.

Both groups performed exercises three times a week for 8 weeks (totaling 24 sessions), completing three exercises each session, with each exercise comprising 10 to 16 repetitions. Rest intervals of 1–2 min were allowed between sets, and 1.5–3 min between exercises. The goal for both programs was to enhance strength through the overload principle. At the end of each main training session, participants engaged in a 5-min cool-down period, including stretches for the hamstrings, gastrocnemius, iliotibial band, and quadriceps.

### 2.5 Statistical analysis

Descriptive data were presented as mean ± standard deviation (SD). The assumption of normality was verified using Shapiro-Wilk test. Levene's test was used to prove the assumption of homogeneity of variances**.** Independent and dependent t-tests were used to compare the variables between groups and between before and after each intervention, respectively. Relative reproducibility was assessed using the two-way random effect interclass correlation coefficient (ICC) [30], interpreted according to Vincent's recommendations: ICC >0.9 indicates high reliability, 0.8 to 0.9 moderate reliability, and <0.8 low reliability [31]. For evaluation of absolute reproducibility, the Standard Error of Measurement (SEM) was calculated using the formula SEM = SD × √ 1 − ICC. In addition, the SEM% was calculated, defined as SEM/(mean of measurements) ∗ 100 [30]. Statistical analyses were conducted using IBM SPSS Statistics for Windows, version 24.0 (IBM Corp., Armonk, NY, USA), with significance set at p < 0.05.

## 3 Results

The individual characteristics of the subjects are presented in Table 1. As shown, there was no significant difference between the two groups (P > 0.05), indicating that the groups were homogeneous in terms of individual characteristics.

**Table 1:** The individual characteristics of subjects.

Variable	Groups		Sig.
	Selective (n = 13)	General (n = 12)	
Age **(years)**	25.4 ± 2.1	26.5 ± 2.3	0.731
Height **(cm)**	166.2 ± 12.7	168.2 ± 13.1	0.817
Weight **(kg)**	67.8 ± 5.9	66.8 ± 5.7	0.936
BMI **(kg/m**^**2**^**)**	23.5 ± 1.9	24.4 ± 2.3	0.619

Data are presented as mean ± standard deviation.

The ICC indicated high values of relative reproducibility, ranging from 0.936 to 0.984 (95 % CI 0.917 to 0.998). Absolute reproducibility expressed as SEM% was 3.6–6.7 % (Table 2).

**Table 2:** Relative and absolute reproducibility statistics for muscles activities and cross-sections.

	ICCs (Level of Reliability)	95 % CI		SEM
		Lower	Upper	
VM RMS	0.936 (High)	0.918	0.966	3.6
VL RMS	0.941 (High)	0.917	0.962	4.2
VM C-S	0.984 (High)	0.967	0.998	6.7
VL C-S	0.982 (High)	0.964	0.995	6.5

VM RMS: Vastus medialis activity; VL RMS: Vastus lateralis activity; VM C-S: Vastus medialis cross-section; VL C-S: Vastus lateralis cross-section; ICCs: Interclass correlation coefficients; 95 % CI: 95 % confidence interval; SEM: Standard error of measurement.

The intergroup differences of the variables are shown in Table 3. According to this table, no significant differences were observed between the groups before the intervention (P > 0.05). However, after the intervention, the activity and cross-section of the vastus medialis, as well as the ratio of the activity and the cross-section of the vastus medialis to the vastus lateralis in the selective group were significantly higher than before the intervention and higher than the general group (P < 0.01). Nevertheless, in the general group, the activity and cross-section of the vastus lateralis were significantly higher than before the intervention and higher than the selective group (P < 0.01).

**Table 3:** The results of intergroup comparison in pre-intervention and post-intervention.

Variable	Phase	Selective	General	Sig.
VM RMS	pre-intervention	23.94 ± 3.86	25.53 ± 3.79	0.391
	post-intervention	^●^∗38.15 ± 6.12	^●^30.22 ± 4.70	0.001
VL RMS	pre-intervention	29.18 ± 4.53	31.40 ± 5.38	0.487
	post-intervention	31.22 ± 4.69	∗^●^40.15 ± 6.16	0.004
VM/VL RMS Ratio	pre-intervention	0.808 ± 0.15	0.803 ± 0.16	0.607
	post-intervention	^●^∗1.215 ± 0.19	0.789 ± 0.16	0.001
VM C-S	pre-intervention	3.28 ± 0.96	3.32 ± 0.99	0.566
	post-intervention	∗^●^5.14 ± 1.29	3.61 ± 1.03	0.001
VL C-S	pre-intervention	1.98 ± 0.47	1.93 ± 0.47	0.719
	post-intervention	2.27 ± 0.62	^●^∗2.85 ± 0.76	0.005
VM/VL C-S Ratio	pre-intervention	1.64 ± 0.41	1.60 ± 0.42	0.518
	post-intervention	^●^∗2.21 ± 0.59	1.59 ± 0.39	0.001

∗ Significant difference with the other group; ● Significant difference with the pre-intervention; P < 0.01. **VM RMS:** Vastus medialis activity; **VL RMS:** Vastus lateralis activity; **VM/VL RMS Ratio:** Vastus medialis/Vastus lateralis activity ratio; **VM C-S:** Vastus medialis cross-section; **VL C-S:** Vastus lateralis cross-section; **VM/VL C-S Ratio:** Vastus medialis/Vastus lateralis cross-section ratio.

**Fig. 2:**
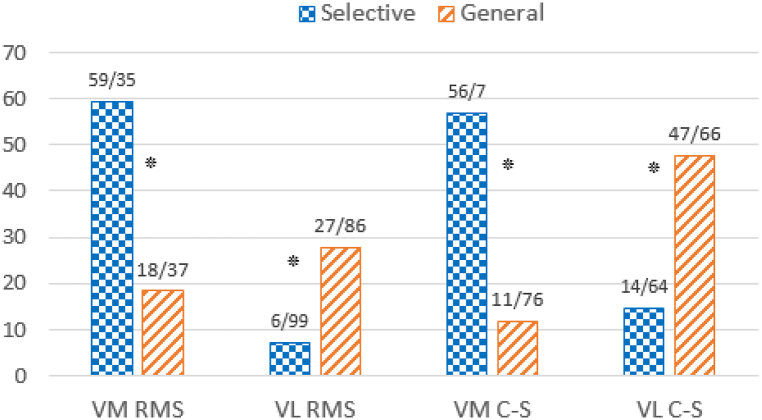
Percentage of intragroup changes in muscles activity and cross-section from pre-to post-intervention. ∗ Significant difference between two groups, P < 0.01.

**Fig. 3:**
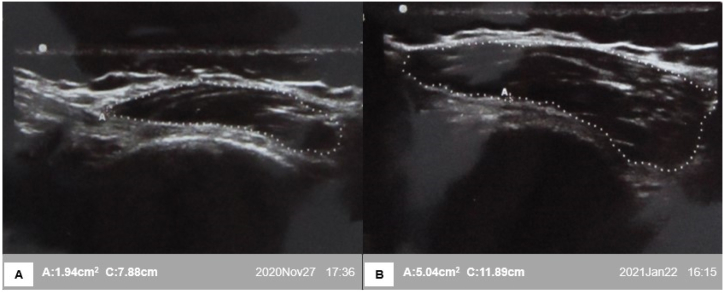
Cross-section of the vastus medialis muscle in one patient from the selective group. a) Pre-intervention. b) Post-intervention.

The percentage of intra-group changes in activity and cross-section of muscles from pre-to- post interventions are shown in Fig. 2. As illustrated, the percentage of intra-group changes in the activity and cross-sectional area of the vastus medialis in the selective group was significantly higher than in the general group (P < 0.01). In contrast, in the general group, the percentage of intra-group changes in the activity and cross-sectional area of the vastus lateralis was significantly higher than in the selective group (P < 0.01). Additionally, Fig. 3 displays the cross-sectional area of the vastus medialis muscle in one patient from the selective group before and after the intervention.

**Fig. 4:**
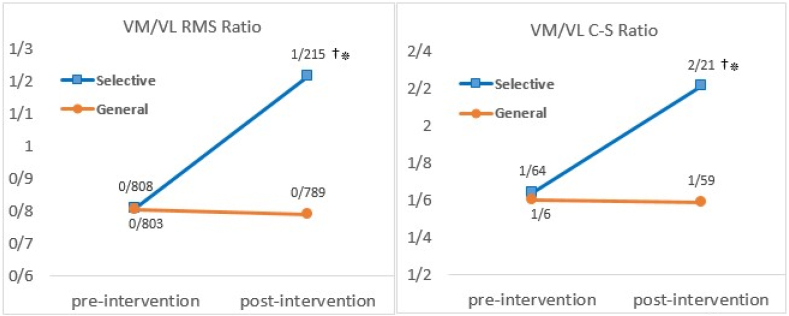
Comparison of the ratios of activity and cross-section of vastus medialis to vastus lateralis. ∗ significant difference between two groups; † Significant difference with pre-intervention. P < 0.01.

In addition, the ratios of activity and cross-section of vastus medialis to vastus lateralis is shown in Fig. 4. This figure indicates that after the intervention, the ratios of activity and cross-sectional area of the vastus medialis to the vastus lateralis in the selective group were significantly higher than before the intervention (P < 0.01) and significantly higher than in the general group (P < 0.01).

## 4 Discussion

The results of this study indicate that in the selective group, the activity of the vastus medialis, the ratio of the vastus medialis activity to that of the vastus lateralis, the cross-sectional area of the vastus medialis, and the ratio of the vastus medialis cross-section to the vastus lateralis all increased significantly from pre-to post-intervention, while the vastus lateralis activity and cross-sectional area showed no significant change. An analysis of intra-group changes reveals a 59.35 % increase in activity and a 56.7 % increase in the cross-sectional area of the vastus medialis in the selective group. These findings demonstrate the significant effect of selective strengthening exercises for the vastus medialis, highlighting their potential to reduce imbalances between the medial and lateral quadriceps components. In contrast, general quadriceps strengthening exercises increased the activity of both the vastus medialis and vastus lateralis (with a greater increase in the vastus lateralis) and enlarged the vastus lateralis cross-section. As a result, such exercises do not correct the imbalance between these two muscles; instead, they may exacerbate it.

These findings are important because researchers have proven that the weakness of the vastus medialis compared to the vastus lateralis [[6], [7], [8], [9]] and the imbalance between the morphology of the vastus medialis and the vastus lateralis muscles [[10], [11], [12], [13]] are the main factors of external malalignment and abnormal patellar tracking during knee joint movements. Also, an atrophy in the vastus medialis and vastus lateralis, with atrophy more evident in the vastus medialis, was reported in patients with patellofemoral pain compared to normal controls [32], and it has been suggested that patellofemoral morphology is altered in adolescents with patellofemoral pain and affects patellar tracking, highlighting the multifactorial etiology of this pain [33]. Despite this information confirming our results, Crouzier et al. stated that there is no difference between adolescents with patellofemoral pain and matched adolescents without knee pain in the distribution of VM and VL torque indices during isometric tasks with 60 of knee flexion [34]. However, in our study (focusing on lateral patellar compression syndrome), the patella was positioned more laterally than in typical patellofemoral pain cases, which may influence or be influenced by the structural and functional characteristics of these muscles. In addition, it has been said that demographic parameters such as height, weight and BMI influence patellofemoral kinematics [35] and the patients of our study had different demographic characteristics compared to the study of Crouzier et al. [34].

The observed increase in vastus medialis activity following isokinetic knee extension exercises in this study may relate to the muscle's length-tension relationship, where maximum external tibial rotation places the vastus medialis under peak tension, maximizing contraction force. Isokinetic exercises, with their constant speed and variable resistance, provide maximum resistance throughout the range of motion, requiring greater motor unit recruitment and an increased muscle firing rate. Repeated exposure to this resistance likely enhances vastus medialis strength and activity. Moreover, our study used a concentric-eccentric cycle; eccentric exercises have been shown to generate more tension [36], primarily due to the passive force from elastic components during eccentric contractions [37]. Overall, isokinetic training improves neuromuscular responses, enhances motor unit coordination, maximizes muscle contraction throughout the range of motion, and promotes muscle fiber recruitment and adaptation across various speeds. These exercises are particularly effective in strengthening fast-twitch fibers [38], which are more prevalent in the vastus medialis compared to the vastus lateralis [39] and hypertrophy in the fast-twitch fibers usually occurs more [40], supporting the results of this study.

Previous studies have sought to design exercises that specifically engage the vastus medialis more than the vastus lateralis to improve patellofemoral joint function [[17], [18], [19],^41^]. However, no exercises have been identified that selectively target the vastus medialis over other quadriceps components. Some research suggests that selective activation of the vastus medialis relative to the vastus lateralis may not be possible [20,21]. Despite this claim, Syme et al. [21]. only evaluated pain, function, and quality of life without assessing the strength and morphology of the vastus medialis and lateralis, which are crucial in such patients. Similarly, Kooiker et al. [20]. did not compare different training programs (e.g., general quadriceps strengthening vs. selective strengthening) but rather concluded in a review that isolated quadriceps strengthening is more effective than advice and information alone for pain reduction and functional improvement. Smith et al. [23]. in a systematic review of electromyographic studies, questioned whether the vastus medialis could be preferentially activated, concluding that strong evidence for this is lacking. Therefore, they recommended that clinicians should not focus on vastus medialis strengthening, in preference to general quadriceps training when rehabilitating patients with patellofemoral disorders, because this may not be possible. Despite the lack of consensus on a gold standard for selective strengthening of the vastus medialis or vastus lateralis [36], the current study's findings provide clinical evidence that isokinetic knee extension exercises with maximum tibial external rotation may selectively strengthen the vastus medialis in young women with lateral patellar compression syndrome.

Despite the important findings, the study's main limitation is the absence of patient-reported outcomes, which could have enabled direct comparison with similar research. Additionally, demographic limitations, such as the age and gender of participants, may impact the generalizability of the findings. Including a healthy control group could further enhance the precision and generalization of the results.

## 5 Conclusion

Based on the findings of this study, traditional quadriceps general strengthening exercises may maintain or even exacerbate the existing imbalance in activity and morphology between the vastus medialis and vastus lateralis muscles. In contrast, isokinetic knee extension exercises performed during the final 30 degrees of the range of motion with maximum external leg rotation can significantly improve the ratios of activity and cross-section of the vastus medialis to the vastus lateralis. These results suggest that, contrary to previous evidence, it is possible to selectively activate and strengthen the vastus medialis muscle independently of other quadriceps components using the specified exercise protocol. Hence, sports medicine clinics and physiotherapy centers are recommended to use such exercises for these patients and those with other similar chronic patellofemoral disorders in order to reduce the imbalance between the vastus medialis and vastus lateralis muscles; An imbalance that seems to be one of the important factors in the patellar malalignment and the pressure applied on its lateral aspect.

## CRediT authorship contribution statement

**Seyyed Hossein Hosseini:** Writing – review & editing, Writing – original draft, Validation, Software, Resources, Project administration, Methodology, Investigation, Formal analysis, Data curation, Conceptualization. **Farzam Farahmand:** Supervision, Resources, Project administration, Methodology, Conceptualization.

## Ethics statement

This study was approved by the Research and Technology Council of Nahavand University (Ethical Code IR. NAHGU.REC.1399.003).

## Data availability statement

Data collected during the trial are available upon request, after de-identification of requesters. data related to the variables mentioned in this research will be made available immediately after publication (with no end date) to researchers who provide a methodologically sound proposal and direct it to hoseini.papers@gmail.com. For access, data requesters must sign a data access agreement.

## Funding

The author(s) reported there is no funding associated with this work.

## Declaration of competing interest

The authors declare that they have no known competing financial interests or personal relationships that could have appeared to influence the work reported in this paper.
